# Student Competency for Midtrimester Obstetrics Scan upon Completion of the Master's Degree in Medical Sonography

**DOI:** 10.1155/2022/2625242

**Published:** 2022-10-27

**Authors:** Surapa Hairunpijit, Surachate Siripongsakun, Chanisa Chotipanich, Pantajaree Hiranrat, Amarin Narkwichean, Wipada Laosooksathit

**Affiliations:** ^1^Sonographer School, Faculty of Health Science Technology, Chulabhorn Royal Academy, Bangkok, Thailand; ^2^Radiological Department of Central Chest Institute of Thailand, Bangkok, Thailand; ^3^Department of Obstetrics and Gynecology, Faculty of Medicine, Srinakharinwirot University, Ongkharak, Nakhon Nayok, Thailand

## Abstract

**Objectives:**

To evaluate the competency of medical sonographer students who have completed training to estimate the gestational age (GA) and perform fetal biometric measurements compared to obstetricians.

**Methods:**

We conducted a cross-sectional observational study at the end of the medical sonographer students' practice sessions. In total, 80 midtrimester (18–28 weeks) pregnant women were recruited, and an ultrasound was performed according to the International Society of Sonography in Obstetrics and Gynecology (ISUOG) guideline. Estimated GA calculated from fetal biometric measurements was compared between medical sonographer students and qualified obstetricians. Subsequently, images were randomly evaluated by maternal-fetal medicine specialists to assess the measurement performance.

**Results:**

There was no significant difference in the estimated GA between the medical sonographer students and obstetricians (mean difference, 0.01 ± 2.92 day, *p* = 0.89). However, there was a significant difference in the measurement of the head circumference (HC) and abdominal circumference (AC) (*p* < 0.001). The overall image quality of the fetal head, abdomen, and femur was considered a good to excellent score (77.5%–80%). There was a perfect and nearly perfect agreement regarding the presence of the placenta previa, adequacy of amniotic fluid, and position of the placenta (*k* = 0.9–1.0).

**Conclusions:**

The medical sonographer students demonstrated competency in GA estimation by fetal biometry measurement similar to obstetricians. However, the quality of the acquired images, according to the ISUOG recommendation, needs improvement, and this should be emphasized in the sonography course curriculum. The results suggest that medical sonographers can relieve obstetricians' workload for ultrasound screening in midtrimester pregnancies.

## 1. Introduction

Appropriate antenatal care is essential for all pregnancies to achieve the best outcomes. Quality antenatal care includes health promotion, prevention, screening, and diagnosis of diseases. To reach the expected quality standard, obstetrics ultrasound has become a significant part of antenatal care; for example, the World Health Organization (WHO) recommends an early obstetrics ultrasound before 24 weeks of gestational age (GA) [1]. The obstetrics ultrasound, such as gestational age estimation and evaluation of the fetal development/well-being, are essential for delivering quality antenatal care. The current obstetrics ultrasound practices mostly follow The International Society of Ultrasound in Obstetrics and Gynecology (ISUOG) recommendation [2, 3].

In many countries, medical sonographers perform scanning to assist specialized physicians. The ultrasound technique is highly operator-dependent; therefore, optimal performance requires careful training, good skills, and experience [4]. In Thailand and some other countries, all obstetrics scans are currently done by either obstetricians or radiologists. As ultrasound examination becomes more routine in antenatal screening, it will have a significant impact on the physician workload. In 2018, a newly established master's degree sonographer training program was established at the new Sonographer School of Chulabhorn College of Medical Science [5]. The aim is to develop sonographers with an enhanced capacity to use ultrasound technology to provide thorough ultrasound services for diagnosis and treatment, including obstetrics scans. With the new training program now established, assessing the competency of the first batch of master's degree students is vital to assessing and improving the program's curriculum. A few previous studies have proven that medical sonographers can perform ultrasound scanning and detect hepatic and biliary lesions with similarity to radiologists [6, 7]. However, there is still no study on the competency evidence of medical sonographers in performing obstetric ultrasound. Thus, our study decided to evaluate the student's competency in terms of obstetric midtrimester scan, i.e., biometric measurement, evaluation of the number of fetuses, placental location, and amniotic adequacy, when compared to obstetricians.

## 2. Methods

This observational study evaluated the sonographer students' competency after completing their obstetrics modules to perform midtrimester ultrasound compared to qualified obstetricians in 80 singleton pregnant women at Srinakharinwirot University Hospital, Thailand. The research was approved by the Ethics Committee for Human Research at the Chulabhorn Research Institute (CRI 073/2562). In the master's degree medical sonography curriculum, students have compulsory training in obstetrics sonography, which consists of two modules: (i) basic obstetric ultrasound, which accounts for 2 credits with 15 hours of didactic lectures, and 30 hours of simulation (and clinical) practice, and an allotment of 45 hours of self-learning; and (ii) obstetric clinical clerkship, which allows a total of 175 hours of obstetrics sonography scanning practice. To complete both modules, students must pass both written and practical examinations. The study evaluated the competency of sonographer students immediately at the end of the obstetric clinical clerkship module (which is in the second year of the 2-year study).

The research was conducted following all relevant international ethical guidelines. The inclusion criteria were women aged ≥20 years old, carrying singleton pregnancies, who visited the antenatal clinic during their second trimester (18–28 weeks). Their gestational age had to be confirmed at least once during a first-trimester ultrasound scan. The exclusion criteria were the presence of a fetal anomaly detected during the scan.

All participants (patients) provided the written informed consent. All the participants were enrolled in the study. Demographic data were collected, including age, gestational age, height, weight, previous pregnancy history, and menstrual period. The participants then underwent two ultrasound scans: the first one conducted by a medical sonographer student and then a second performed by a qualified on-duty obstetrician. Two sonographer students (only 2 students enrolled in the course in the first academic year) were involved in this study. For each participant, both a sonographer student and the obstetricians completed the level 1 ultrasound, including measuring the biometric parameters, placental location, and performing an estimation of the amniotic fluid according to the ISUOG guideline. The biometric parameters included the biparietal diameter (BPD), head circumference (HC), abdominal circumference (AC), and femur length (FL) [3]. The sonographer students performed the measurement three times for reproducibility assessment.

**Figure 1:**
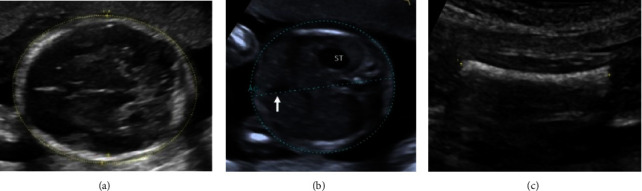
Fetal biometric plane and measurement: (a) measurement of the head circumference (HC), where an ellipse is drawn around the outer calvarium and biparietal diameter (BPD), showing the measurement diameter from the outer to the inner table of the parietal bone (cross mark measurement); (b) measurement of the abdominal circumference (AC) in the transverse abdominal view approach showing a symmetrical plane with the presence of the fluid-filled stomach (ST) and portal sinus (arrow); (c) measurement of the femur length (FL) in a longitudinal view showing the presence of the distal and proximal ends of the diaphysis, in which the measurement points should be placed at the midpoint of each diaphyseal end (cross mark measurement).

Information regarding the correct image planes/measurement techniques and criteria for the best-quality biometric images are presented in Figure 1 and Table 1, respectively. GA estimation was performed according to the Hadlock formula [8], with the placental site and adequacy of the amniotic fluid recorded. Also, three sonographic images demonstrating (i) the fetal head on the transthalamic plane, (ii) a transverse section of the fetal abdomen (as circular as possible), and (iii) the femur diaphysis length recorded in all participants during the sonographer students' scans. Subsequently, 60 images from 20 studies were randomly selected for evaluation (and marking) to assess the imaging quality by a maternal-fetal medicine specialist (WL) using the ISUOG guideline assessment of fetal biometry and growth (Table 1) [2, 9, 10]. The sonographer students used the LOGIQ S7 system (GE Healthcare, Austria), while the obstetrician used the Voluson E6 system (GE Healthcare, Austria) for the scans in the study.

### 2.1. Statistical Analysis

Demographic data are presented using descriptive statistics. The mean gestational age and fetal biometry measurements between the medical sonographer students and obstetricians were compared using the Wilcoxon signed-rank test statistic. There were only two students in the first batch of course students. Thus, two sonographer students performed the scans in the present study. The interobserver variability and mean difference in GA by each sonographer student and the obstetricians were analyzed using the Mann–Whitney *U* test. The intraobserver reliability of the fetal biometry measurements was calculated using the intrasubject coefficient of variation (%CV) [11]. The correlation agreement between the medical sonographer students and obstetricians for assessing the placenta site, the presence of placenta previa, and the adequacy of amniotic fluid was assessed by Fleiss and Cohen's Kappa statistics, respectively. The Kappa coefficient was determined as follows: 0 as agreement equivalent to chance; 0.01–0.20 as slight agreement; 0.21–0.40 as fair agreement; 0.41–0.60 as moderate agreement; 0.61–0.80 as substantial agreement; 0.81–0.90 as near-perfect agreement; and 1.00 as perfect agreement.

Statistical analysis was performed utilizing the IBM Statistical Package for the Social Sciences (SPSS) version 20 (IBM, Inc., New York, NY), and significance was defined as a *p* value <0.05.

## 3. Results

In total, 81 pregnant women were enrolled in the study, with one excluded due to fetal cleft lip and cleft palate, and two were missing some measurement data. The participants' average age and BMI were 29.55 ± 4.62 years old and 23.94 ± 3.85 kg/m^2^, respectively. Their average gestational age on the scan date was 20.8 ± 7.53 weeks. The participants' demographic data are presented in Table 2.

There was no significant difference in estimated gestational age (biometric calculation) measured between the medical sonographer students and obstetricians (mean difference of GA measured = 0.01 ± 2.92 days, *p* = 0.89). Regarding each biometric parameter, the BPD and FL measurements were also not significantly different (mean difference BPD = 0.39 ± 1.94 mm, *p* = 0.07; FL = 0.12 ± 1.73 mm, *p* = 0.94). Nonetheless, the circumferential measurements (HC and AC) were measured as slightly larger by the medical sonographer students when compared to the obstetricians (mean difference = 4.08 ± 5.88 mm, *p* < 0.001, and 2.86 ± 7.23, *p* < 0.001, respectively). The mean differences in fetal biometry and estimated GA and comparisons between the medical sonographer students and obstetricians are shown in Table 3. Overall, interobserver variation was not observed when comparing the mean difference in GA determined by the medical sonographer students and obstetricians (*p* = 0.39). Also, the interobserver variability between sonographers was not significantly different for the BPD, HC, and AC measurements (*p* = 0.78, 0.20, and 0.14), and there was only a significant difference in FL measurement (*p* < 0.001). Besides, the intraobserver variation for the two medical sonographer students' measurements showed no significant difference in BPD, HC, AC, and FL for three repeated measurements. The intrasubject coefficient of variations (%CV) for BPD, HC, AC, and FL by medical sonographer student number 1 was 1.68%, 1.28%, 1.54%, and 1.61%, respectively, and for medical sonographer student number 2 was 1.53%, 1.43%, 1.96%, and 2.43%, respectively. We observed total agreement with placenta previa detection and evaluation of the amniotic fluid volume between the medical sonographer students and obstetricians (Kappa = 1). A near-perfect agreement on the determination of the placental location was also demonstrated (*k* = 0.9).

Regarding the assessment of the students' skills, the overall average scores between 77.5% and 80% achieved across all the biometric parameters regarding the sonographer students' performance in terms of image capture and measurement quality. Nonetheless, breakdown analysis demonstrated some student defects in (i) obtaining a symmetrical abdominal plane (45%), (ii) obtaining an adequate fetal head plane showing the thalami (55%), and (iii) ensuring the captured fetal part was more than half the total image (AC 60%, FL 55%) (Table 4).

## 4. Discussion

The study demonstrated that the competency of medical sonographer students, after completing the obstetrics module of the master's medical sonography degree at the Sonographer School, was comparable to that of qualified obstetricians in terms of gestational age estimation and evaluation of the placental location and amniotic fluid adequacy during the midtrimester screening scan. A breakdown of the skills assessment regarding gathering appropriate images and performing measurements correctly showed a satisfactory outcome, with an overall average achievement of more than 70% for all the biometric measurements (BPD, HC, AC, and FL). However, there was still room for improvement. The findings also supported the students' competency in detecting abnormalities related to biometry, such as fetal growth restriction, or life-threatening maternal/fetal conditions, such as placenta previa and oligohydramnios [12, 13]. Hence, the findings from the present study provide confidence that medical sonographers are capable and can play a substantial role in obstetrics scans.

This study was about competency assessment in an actual clinical setting regarding gestational age estimation and placental location. The study primarily supports the potential of sonography training, showing sonographers can be trained to a certain level of expectation, depending on the training program's quality. In current obstetric practice, the World Health Organization (WHO) recommends a routine ultrasound scan before 24 weeks in all pregnant individuals [1]. The Royal Thai College of Obstetricians and Gynecologists has echoed the WHO recommendation, though only stating that the practice is suggested, acknowledging the current limitations in national resources. If the routine second trimester ultrasound policy is to be applied, the coverage would be questioned because all obstetric scans are mainly performed by obstetricians and a small proportion by radiologists. The potential workloads would, hence, be enormous, but it is believed that sonographers could significantly help alleviate this. Nonetheless, since the career has not yet been formalized in Thailand and as the sonography course is only just newly established, student competency assessment is necessary.

Our obstetric modules consist of both lectures and practical sessions. Once they pass the knowledge exam, the students are required to undergo 8 weeks of clinical practice, expecting to scan at least 40 cases, both first and second trimester scans. The present study was designed to evaluate the students' critical skills in terms of biometric measurements and evaluate the placental site and amniotic fluid volume, which are essential elements of the second trimester scan. Such evaluation should be performed prospectively on a regular basis. The current research can preliminarily guarantee the performance of sonographers to the obstetrics community. With support from obstetricians and radiologists, either on-site or remotely, sonographers could help screen prenatal populations for obstetric and neonatal risks, with the potential to improve outcomes at delivery and provide site-specific epidemiologic data that can be used to develop new healthcare provision strategies [14].

Concerning each parameter measurement, there were significant differences between the sonographer students and obstetricians for the HC and AC measurements. These differences could be explained by the fact that the HC and AC measurements are two-dimensional measurements [3] that need multiple precise caliper placements. Thus, HC and AC measurements tend to be more prone to errors than one-dimensional measurements, such as BPD and FL. We observed errors similar to those reported in the study done by Neufeld et al. [15]. Although the differences in AC and HC measurements have no clinical impact on gestational age estimation, this should be emphasized in the sonography training in the following years. On the contrary, FL measurement was observed to be the least reliable between the two sonographer students, which was a similar finding to that in Sarris's study, which observed an 11.1% interobserver variation [16]. This could be because the fetal leg has the most mobility during scanning, and its position is prone to change, resulting in a lower repeatability of the measurement.

To identify areas for teaching/learning improvement in the following years, image analysis was performed by a maternal-fetal medicine specialist to assess the skills of the sonographers. It was shown that the average range of achievement in obtaining an optimal image of the head, abdomen, and femur planes was between 77.5% and 80.0%. We observed that getting a symmetrical plane of the fetal abdomen was the least achieved (45%). Obtainment of the thalamic plane and performing FL image magnification correctly was the second lowest achieved (55%). By considering the image quality with the measurement results, achieving (i) the plane showing the thalami, (ii) the symmetrical plane of the AC, and (iii) correct image magnification were among the weakest skills of the medical sonographers. These findings were caused by either improper measurement or poor image landmarks for precise placement of the calipers. To improve the sonographer students' competency, the course needs to help the students develop multiple sonographic skills, including hand-eye coordination, image optimization, and anatomical recognition, which need to be built up over the course of the study [17, 18]. In addition, in terms of fetal anomaly scans and teleconsultation, the precision of the image or video clip collection for a certain fetal organ is essential. The findings from this study provide some basic information concerning the areas in the sonography course curriculum that should be enhanced to raise the standards of the students.

Medical sonographer students who have completed clinical training in obstetrics sonography were proved to have a certain competency level in midtrimester ultrasound, including confirming the gestational age estimation, placental location, and amniotic fluid adequacy. However, the quality of the acquired images, according to the ISUOG recommendation, requires some improvement, and this should be emphasized in the sonography course curriculum. We believe that medical sonographers can alleviate the workload of obstetricians in ultrasound screening in midtrimesters in the near future.

## Figures and Tables

**Table 1:** Criteria for the score-based objective evaluation of the quality of the biometric images.

		Diagram	Ultrasound image
BPD & HC	Symmetrical plane	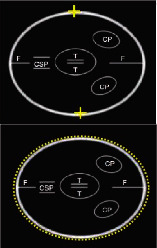	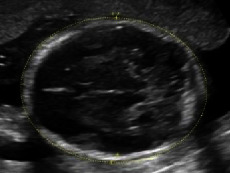
	Plane showing the thalami		
	Plane showing the cavum septi pellucidi		
	Cerebellum not visible		
	Head plane occupying more than half the total image size		
	Calipers and dotted ellipse placed correctly		

AC	Symmetrical plane	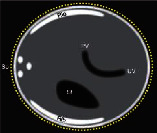	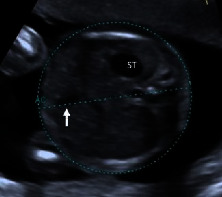
	Plane showing the portal sinus		
	Plane showing the stomach bubble		
	Kidneys not visible in the total image		
	Abdomen plane occupying more than half the total image size		
	Calipers and dotted ellipse placed correctly		

FL	Both ends of bone clearly visible	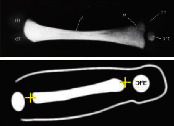	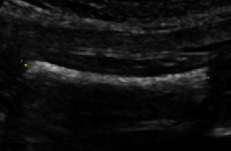
	Femur occupying more than half the total image		
	<45° angle to the horizontal		
	Calipers placed correctly		

**Table 2:** Participants' demographic data.

	*N* = 80
Age (years)^*∗*^	29.55 ± 4.62
Weight (kg)^*∗*^	60.96 ± 11.48
Height (cm)^*∗*^	1.59 ± 0.06
Gestational age at study date^*∗*^ (weeks/days)	20 w 5 d ± 7.5 d
Body mass index (BMI) (kg/m^2^)^*∗*^	23.94 ± 3.85
Normal (BMI < 23): *N* (%)	35 (44.87%)
Overweight (BMI at 23.5–27): *N* (%)	25 (32.05%)
Obesity (BMI > 27–30): *N* (%)	12 (15.38%)
Morbid obesity (>30): *N* (%)	6 (7.70%)
Gravidarum	
Nulliparous: *N* (%)	23 (29.5%)
Multiparous: *N* (%)	55 (70.5%)
Previous caesarean section: *N* (%)	66 (84.6%)

^*∗*^Mean ± standard deviation.

**Table 3:** Comparison of the biometric measurements performed by the sonographer students and the obstetricians.

	Obstetricians	Mean difference	*P* value	Mean difference		*P* value
	(*n* = 80)			Sonographer 1	Sonographer 2	
BPD (mm ± SD)	47.92 ± (3.89)	0.39 ± (1.94)	0.07	0.37 ± (1.95)	0.44 ± (1.95)	0.78
HC (mm ± SD)	180.54 ± (13.66)	4.08 ± (5.88)	<0.001	3.48 ± (5.64)	6.32 ± (6.37)	0.20
AC (mm ± SD)	157.35 ± (14.66)	2.86 ± (7.23)	<0.001	2.24 ± (6.77)	5.13 ± (8.58)	0.14
FL (mm ± SD)	33.77 ± (3.53)	0.12 ± (1.73)	0.94	0.25 ± (1.39)	1.50 ± (2.14)	<0.001
Estimated GA by ultrasound (days ± SD)	20 w4 d ± (8.05)	0.01 d ± (2.92)	0.89	0.20 ± (2.81)	0.64 ± (3.31)	0.39

**Table 4:** Quality of the biometric ultrasound images.

		*n* = 20 (%)	Overall average achievement
Head	Symmetrical plane	16 (80%)	4.8/6 (80%)
	Plane showing the thalami	11 (55%)	
	Plane showing the cavum septi pellucidi	16 (80%)	
	Cerebellum not visible	20 (100%)	
	Head plane occupying more than half the total image size	17 (85%)	
	Calipers and dotted ellipse placed correctly	16 (80%)	

Abdomen	Symmetrical plane	9 (45%)	4.65/6 (77.5%)
	Plane showing the portal sinus	14 (70%)	
	Plane showing the stomach bubble	19 (95%)	
	Kidneys not visible on total image	19 (95%)	
	Abdomen plane occupying more than half the total image size	12 (60%)	
	Calipers and dotted ellipse placed correctly	20 (100%)	

Femur	Both ends of bone clearly visible	16 (80%)	3.15/4 (78.75%)
	Femur occupying more than half the total image	11 (55%)	
	<45° angle to the horizontal	20 (100%)	
	Calipers placed correctly	16 (80%)	

## Data Availability

The data used to support this study are available from corresponding author upon request.

## References

[1] Tunçalp Ӧ., Pena-Rosas J., Lawrie T. (2017). WHO recommendations on antenatal care for a positive pregnancy experience—going beyond survival. *BJOG: An International Journal of Obstetrics and Gynaecology*.

[2] Salomon L. J., Alfirevic Z., Da Silva Costa F. (2019). ISUOG Practice Guidelines: ultrasound assessment of fetal biometry and growth. *Ultrasound in Obstetrics and Gynecology*.

[3] Salomon L. J., Alfirevic Z., Berghella V. (2011). Practice guidelines for performance of the routine mid-trimester fetal ultrasound scan. *Ultrasound in Obstetrics and Gynecology*.

[4] Bello V., Nicastro I., Barletta V. (2013). Professional education, training and role of the cardiac sonographer in different countries. *Journal of Cardiovascular Echography*.

[5] Siripongsakun S. (2020). Sonographer school, HRH princess Chulabhorn college of medical science: the first step of the sonographer system in Thailand. *The ASEAN Journal of Radiology*.

[6] Tangruangkiat S., Phonlakrai M., Ritlumlert N., Siripongsakun S., Vidhyarkorn S., Thitisitthikorn W. (2018). Ultrasonography detection of liver lesions: a pilot comparison study between radiologists and sonographers. *Medical Journal of the Medical Association of Thailand*.

[7] Dawkins A., George N., Ganesh H. (2017). Radiologist and sonographer interpretation discrepancies for biliary sonographic findings: our experience. *Ultrasound Quarterly*.

[8] Hadlock F. P., Deter R. L., Carpenter R. J., Park S. K. (1981). Estimating fetal age: effect of head shape on BPD. *American Journal of Roentgenology*.

[9] Benson C. B., Doubilet P. M. (2017). Fetal biometry and growth. *Callen’s Ultrasonography in Obstetrics and Gynecology*.

[10] Salomon L. J., Bernard J. P., Duyme M., Doris B., Mas N., Ville Y. (2006). Feasibility and reproducibility of an image-scoring method for quality control of fetal biometry in the second trimester. *Ultrasound in Obstetrics and Gynecology*.

[11] Reed G. F., Lynn F., Meade B. D. (2002). Use of coefficient of variation in assessing variability of quantitative assays. *Clinical and Vaccine Immunology*.

[12] Dashe J. S., Hoffman B. L. (2017). Ultrasound evaluation of the placenta, membranes, and umbilical cord. *Callen’s Ultrasonography in Obstetrics and Gynecology*.

[13] Magann E. F., Sandlin A. T. (2017). Amniotic fluid volume in fetal health and disease. *Callen’s Ultrasonography in Obstetrics and Gynecology*.

[14] Greenwold N., Wallace S., Prost A., Jauniaux E. (2014). Implementing an obstetric ultrasound training program in rural Africa. *International Journal of Gynecology & Obstetrics*.

[15] Neufeld L. M., Wagatsuma Y., Hussain R., Begum M., Frongillo E. A. (2009). Measurement error for ultrasound fetal biometry performed by paramedics in rural Bangladesh. *Ultrasound in Obstetrics and Gynecology*.

[16] Sarris I., Ioannou C., Chamberlain P. (2012). Intra-and interobserver variability in fetal ultrasound measurements. *Ultrasound in Obstetrics and Gynecology*.

[17] Tolsgaard M. G. (2018). A multiple-perspective approach for the assessment and learning of ultrasound skills. *Perspectives on Medical Education*.

[18] Tolsgaard M. G., Todsen T., Sorensen J. L. (2013). International multispecialty consensus on how to evaluate ultrasound competence: a Delphi consensus survey. *PLoS One*.

